# A Rare Case of Breast Mucosa-Associated Lymphoid Tissue (MALT) Lymphoma With Amyloid Deposition in a Patient With Sjogren’s Syndrome

**DOI:** 10.7759/cureus.34286

**Published:** 2023-01-27

**Authors:** Hussain Dalal, Arlene Yu, Crystal Antoine-Pepeljugoski

**Affiliations:** 1 Internal Medicine, Nuvance Health, Poughkeepsie, USA; 2 Hematology and Medical Oncology, Albert Einstein College of Medicine, Jacobi Medical Center, Bronx, USA

**Keywords:** serum protein electrophoresis (spep), amyloid, sjogrens syndrome, primary breast cancer, mucosa-associated lymphoid tissue (malt) lymphoma

## Abstract

Marginal zone B-cell lymphoma of mucosa-associated lymphoid tissue (MALT) of the breast with amyloid deposits is a very rare cause of breast malignancy. Patients who carry a diagnosis of Sjogren’s syndrome (SS) have a 5-10% lifetime risk of developing non-Hodgkin lymphoma with MALT lymphoma as the most common histologic subtype. Our case highlights the importance of routine screening mammography in the early detection of such unusual malignancies, and further interventions needed to diagnose and appropriately manage breast MALT lymphoma.

## Introduction

Sjogren’s syndrome (SS) is an autoimmune disease of the exocrine glands that tends to mostly affect the salivary and lacrimal glands. Common clinical symptoms in patients with SS are kerato-conjunctivitis sicca, xerostomia, angular cheilitis, and a host of other symptoms relating to abnormalities in the exocrine glands. Patients with SS have a 5-10% lifetime risk of developing non-Hodgkin lymphoma [1] with marginal zone B-cell lymphoma of mucosa-associated lymphoid tissue (MALT) as the most common histologic subtype. In fact, SS patients have a 1000-fold increased risk of parotid gland MALT lymphoma as compared to the general population [2]. Although the association of MALT lymphoma in patients with SS is well-documented in organs like the stomach and lungs, association with breast MALT lymphoma is exceedingly rare. Belfeki et al. reported a case of breast MALT lymphoma with amyloid deposition in a patient with a long-standing history of Sjogren’s syndrome [3]. 

## Case presentation

**Figure 1:**
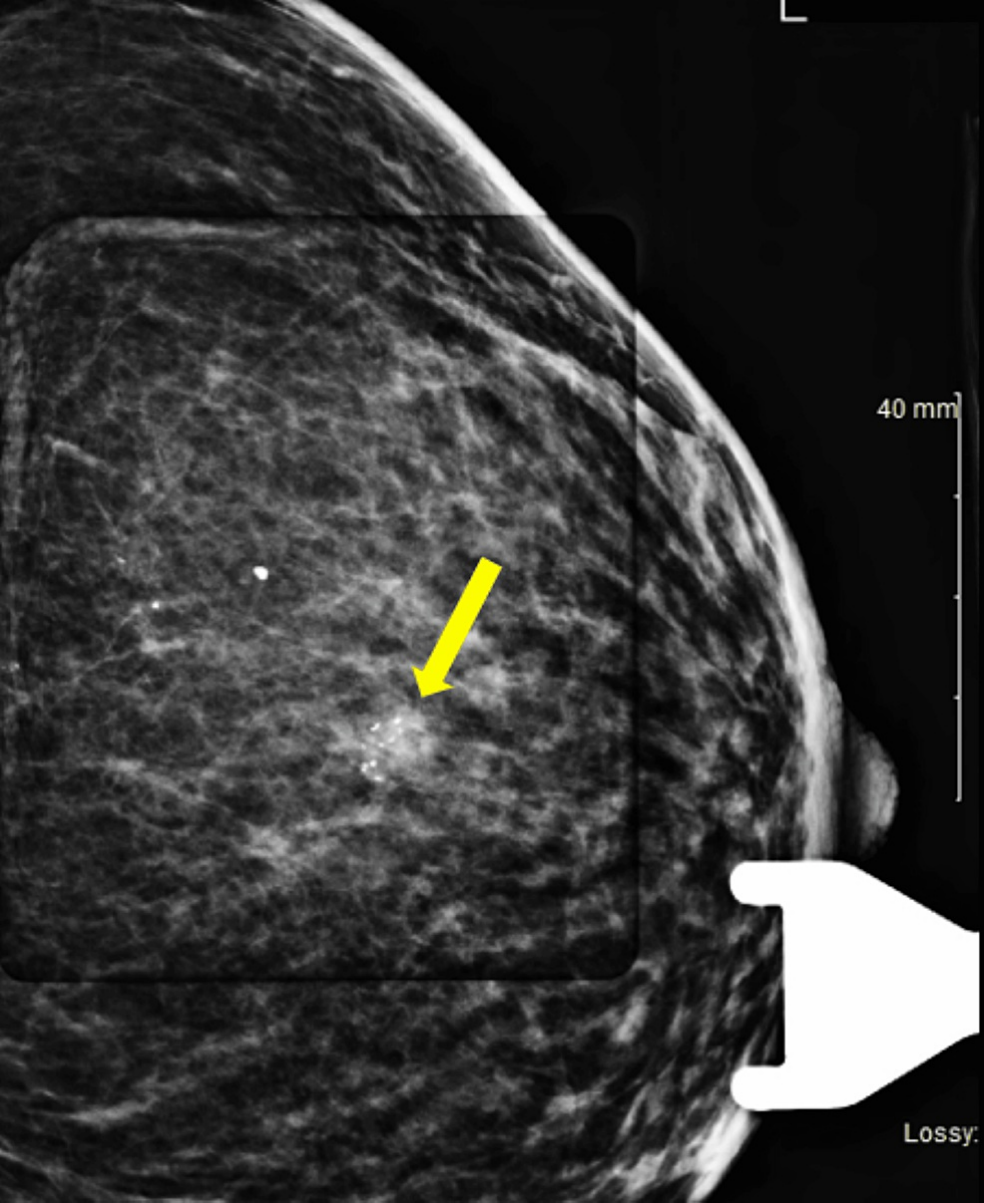
Breast mammogram showing coarse calcifications

A 70-year-old female with a history of SS with extra glandular manifestations of arthritis and dacryocystitis as well as Stage 1 renal cell carcinoma post left radical nephrectomy presented to the oncology clinic after a suspicious routine screening mammogram. Routine mammography was evident for coarse calcifications (Figure 1) in the 12 o’clock position in the middle third of her left breast. The patient denied any symptoms of nipple discharge, breast discoloration, breast pain, axillary pain or swelling, and weight loss. Prior screening mammograms were unremarkable. Her family history was notable for prostate cancer in her father, with no other family history of cancer. Social history was negative for smoking or alcohol use. 

**Figure 2:**
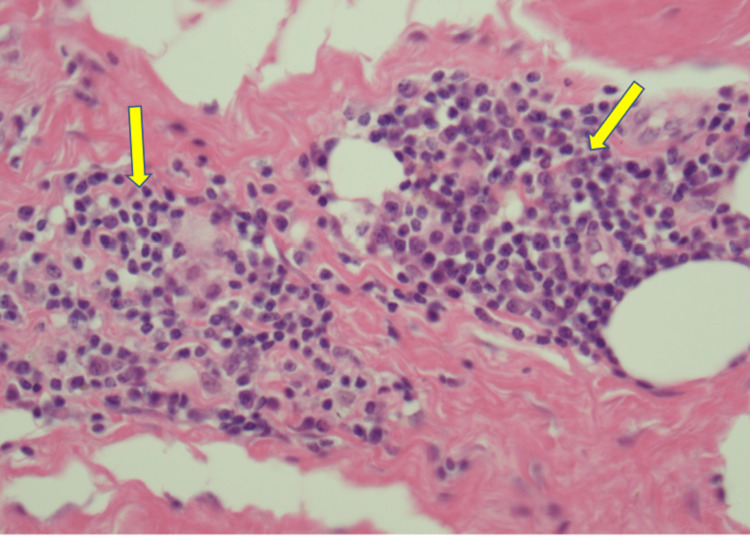
Peri-ductal and peri-vascular lymphoplasmacytic invasion (20X, Yellow arrows)

**Figure 3:**
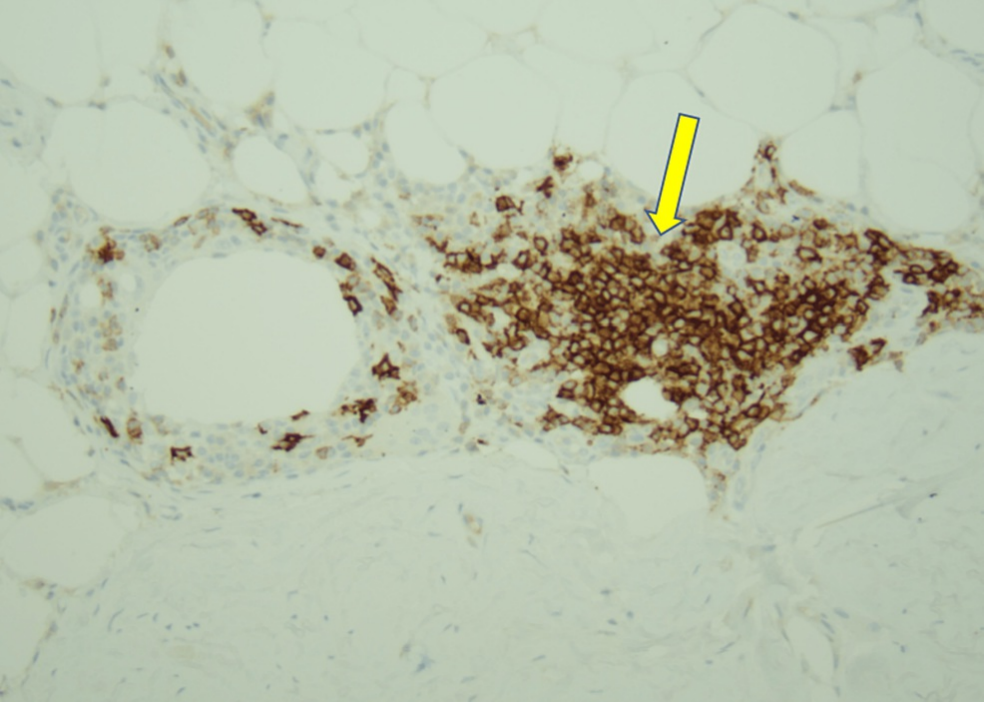
CD20 B lymphocyte stain showing B lymphocytes infiltrating the ductal epithelium (20X, Yellow arrow)

**Figure 4:**
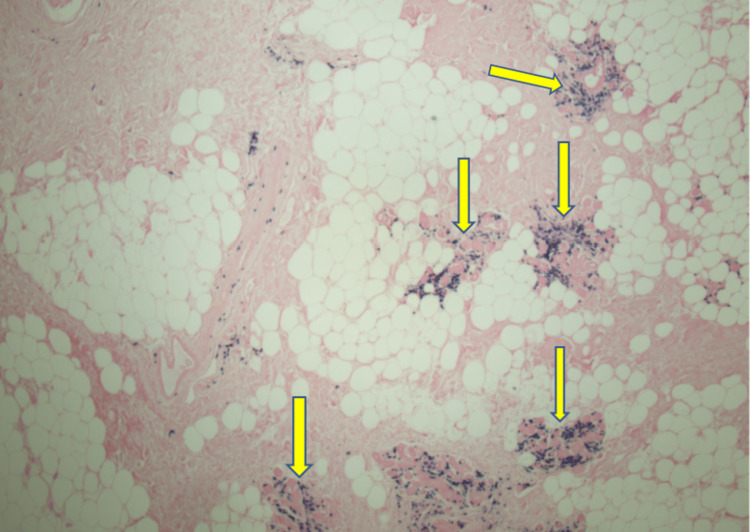
Amyloid deposits (Yellow arrows) in the breast ductal epithelium with foreign body giant cells trying to clear it (4X)

**Figure 5:**
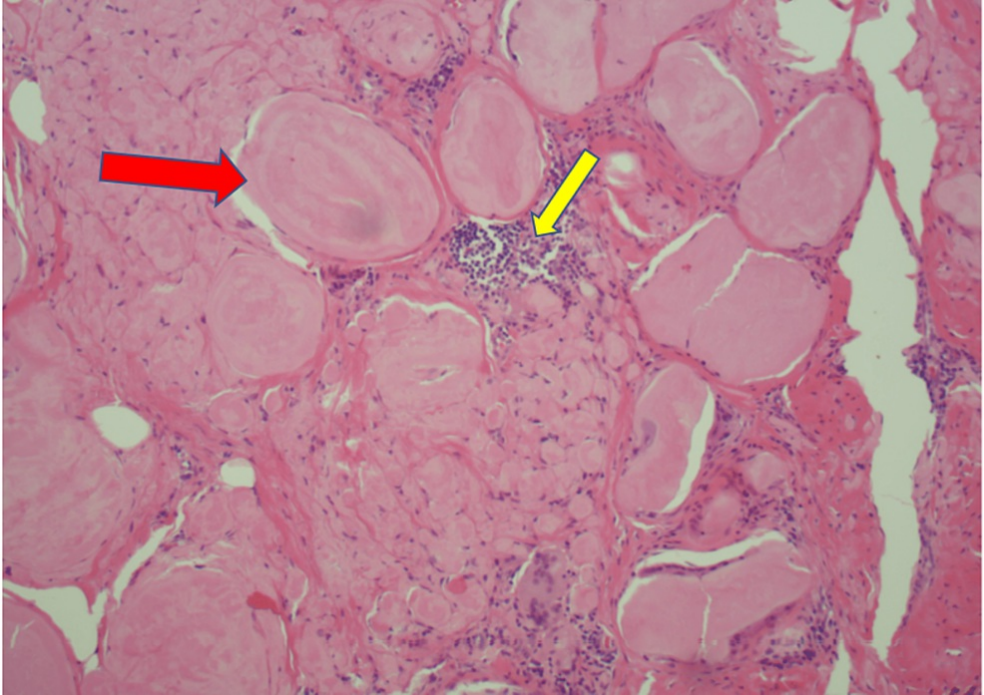
Amyloid deposition (Yellow arrow) with foreign body giant cells (red arrow) trying to clear it (20X)

**Figure 6:**
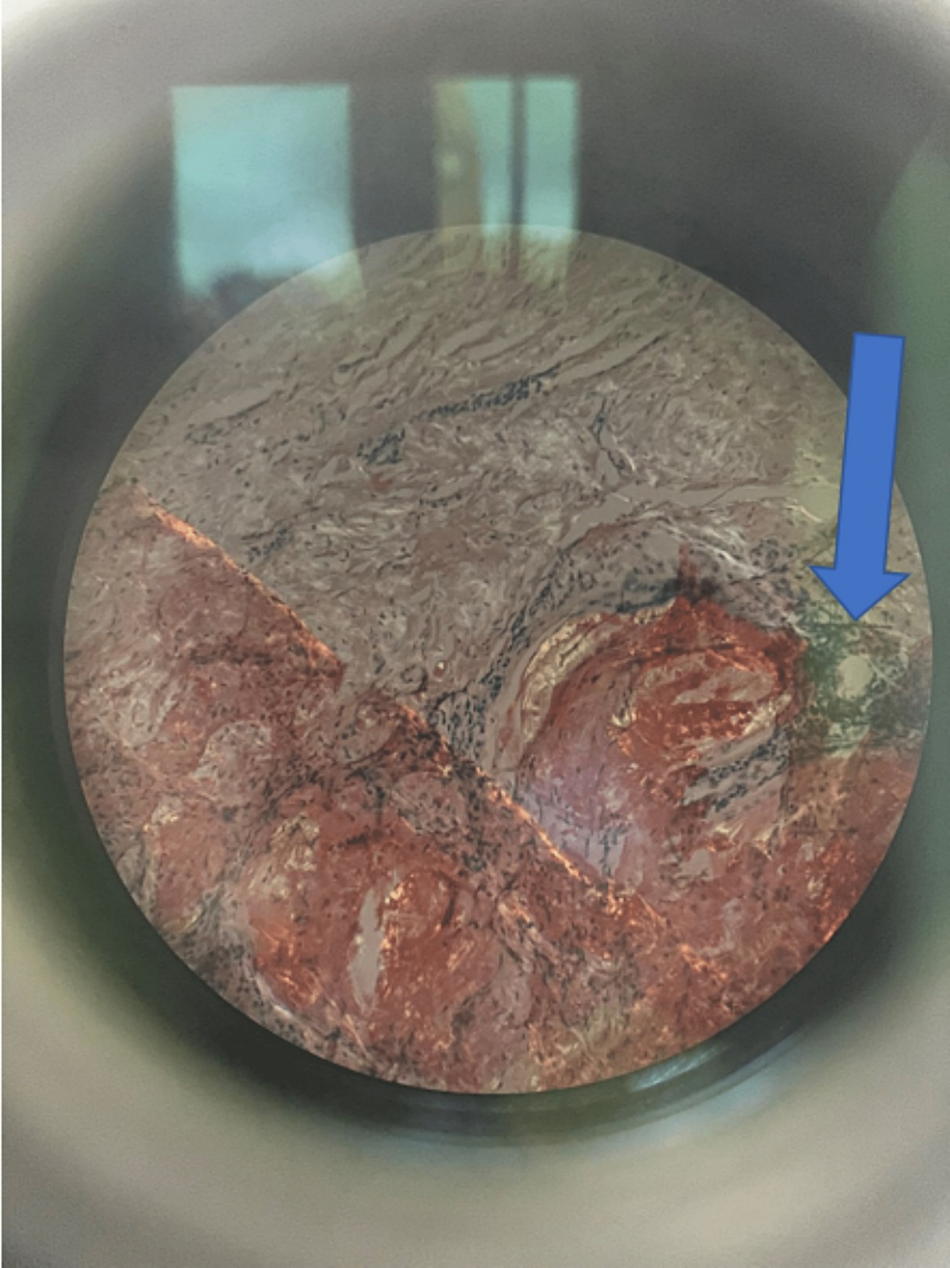
Amyloid deposition in breast ductal epithelium as classically seen in polarized lens with apple green birefringence (Blue arrow)

The patient underwent stereotactic core biopsy and pathology was consistent with marginal zone B cell lymphoma MALT type with light chain restricted plasma cells and amyloid deposits (Figures 2-6). 

Serum immunofixation electrophoresis showed a trivial elevation of kappa/lambda ratio, polyclonal gammopathy, and no monoclonal (M)-protein spike. PET/CT scan did not show any fluorodeoxyglucose (FDG) avidity even in the primary site of the breast. Upper GI endoscopy was negative for co-existing gastrointestinal MALT lymphoma with a negative *Helicobacter pylori* status. 

The patient was referred to the breast clinic where she underwent a successful left breast lumpectomy with a final path diagnosis of MALT lymphoma and negative margins. 

## Discussion

MALT lymphomas are extranodal B-cell lymphomas that are commonly seen in the GI tract (stomach, spleen, etc) and head and neck region. MALT lymphomas involving the breast are rare (<0.5% of all breast malignancies) owing to the paucity of mucosa-associated lymphoid tissue in the breast [3]. Fortunately for our patient, she only had a disease process confined to her breast as evidenced by the negative PET scan as well as an unremarkable GI workup.

As previously noted, SS patients are at a very high risk of MALT lymphomas, but they usually present at sites of submandibular and parotid glands, nasopharynx, thyroid, lungs, and stomach [3]. One of the postulated theories is that MALT lymphoma in these patients is usually characterized by the expansion of B-cells with abnormal expression of CCR5 and CD43 by the infiltrating B cells [3]. However, studies are in progress to determine the exact mechanism of how patients with SS become predisposed to developing lymphomas.

Most patients with MALT lymphoma usually present at an early clinical stage and the disease process is usually amenable to surgery, radiotherapy, or even observation [4]. For example, the standard of care for patients with gastric MALT lymphoma, which is commonly caused by *H. pylori*, is the eradication of *H. pylori*. MALT lymphoma is generally a slow-growing malignancy; however, recurrences and systemic spread can be seen despite maximal therapy [4]. For our patient, given the low grade of her disease and a solitary lesion, surgical resection was deemed to be the appropriate treatment. She continues to have screening mammography for ongoing surveillance.

Amyloid deposition is a rare complication of MALT lymphomas. It has been hypothesized that the plasmacytoid cells in the tumor milieu produce immunoglobulin light chains that are eventually deposited as amyloid [4]. Zhang et al. described two discrete syndromes of lymphoma-associated AL amyloidosis: systemic and peritumoral. Systemic amyloidosis along with high levels of M-protein and multi-organ involvement were commonly associated with lymphoplasmacytic lymphoma (as noted in Figure 2 of pathology images). In contrast, peri-tumoral amyloid deposits, low or undetectable M-protein, and single-organ involvement were generally associated with MALT lymphoma [4]. This was further reported as part of a case series where 20 cases of MALT lymphoma were noted with amyloid deposits at the internal site of the lymphoma and 19 out of 20 cases showed localized peritumoral amyloidosis [5]. Peritumoral amyloidosis was evident in our patient.

Until a more defined pathogenesis and disease prevention strategy is described, a high index of suspicion for the development of MALT lymphomas in SS patients is of utmost importance. Our case also highlights the unusual involvement of the breast with MALT lymphoma in a patient with long-standing SS. Furthermore, it showcases the usefulness of screening mammography in the early detection of this unusual breast malignancy.

## Conclusions

Patients with a long-standing history of autoimmune disorders like SS are at a higher risk of lymphomas. Breast MALT lymphoma is a very rare entity in patients with SS. These patients are also at risk of associated amyloidosis with these malignancies. Fortunately, MALT lymphoma of the breast is usually an indolent malignancy and patients have quite a few treatment options including surveillance, surgery, and/or radiation therapy. Regular screening mammography in this patient population is of paramount importance in detecting these unusual malignancies.
